# Immunization of Domestic Ducks with Live Nonpathogenic H5N3 Influenza Virus Prevents Shedding and Transmission of Highly Pathogenic H5N1 Virus to Chickens

**DOI:** 10.3390/v10040164

**Published:** 2018-03-31

**Authors:** Alexandra Gambaryan, Ilya Gordeychuk, Elizaveta Boravleva, Natalia Lomakina, Ekaterina Kropotkina, Andrey Lunitsin, Hans-Dieter Klenk, Mikhail Matrosovich

**Affiliations:** 1 Chumakov Federal Scientific Center for Research and Development of Immune-and-Biological Products of the Russian Academy of Sciences, premises 8, building 1, Village of Institute of Poliomyelitis, Settlement “Moskovskiy”, 108819 Moscow, Russia; al.gambaryan@gmail.com (A.G.); e@boravlev.mccme.ru (E.B.); nflomakina@yandex.ru (N.L.); ekropotkina@list.ru (E.K.); 2 Institute for Translational Medicine and Biotechnology, Sechenov First Moscow State Medical University, 8 Trubetskaya St., 119991 Moscow, Russia; 3 Federal Research Center for Virology and Microbiology, Bld. 1 Academic Baculov St., 601125 Settl. Volginsky, Vladimir Region, Russia; lunicyn@mail.ru; 4 Institute of Virology, Philipps University, Hans-Meerwein-Str. 2, 35043 Marburg, Germany; klenk@staff.uni-marburg.de (H.-D.K.); matrosov@staff.uni-marburg.de (M.M.)

**Keywords:** influenza virus A, H5N1, poultry vaccine, transmission and environmental control

## Abstract

Wild ducks are known to be able to carry avian influenza viruses over long distances and infect domestic ducks, which in their turn infect domestic chickens. Therefore, prevention of virus transmission between ducks and chickens is important to control the spread of avian influenza. Here we used a low pathogenic wild aquatic bird virus A/duck/Moscow/4182/2010 (H5N3) for prevention of highly pathogenic avian influenza virus (HPAIV) transmission between ducks and chickens. We first confirmed that the ducks orally infected with H5N1 HPAIV A/chicken/Kurgan/3/2005 excreted the virus in feces. All chickens that were in contact with the infected ducks became sick, excreted the virus, and died. However, the ducks orally inoculated with 10^4^ 50% tissue culture infective doses of A/duck/Moscow/4182/2010 and challenged 14 to 90 days later with H5N1 HPAIV did not excrete the challenge virus. All contact chickens survived and did not excrete the virus. Our results suggest that low pathogenic virus of wild aquatic birds can be used for prevention of transmission of H5N1 viruses between ducks and chickens.

## 1. Introduction

Highly pathogenic avian influenza viruses (HPAIV) represent a significant problem in developing countries of Asia and Africa. The situation is particularly alarming in Egypt, where 136 people developed the disease and 39 died of avian influenza virus infection in 2015 [1]. New reassortants of HPAIV with hemagglutinin (HA), polymerase basic protein 2 (PB2) and nucleoprotein (NP) genes from H5N1 viruses emerged in recent years, in particular, H5N3, H5N8 and H5N2 viruses detected in China and North America [2,3].

It is generally accepted that HPAIV are deadly to chickens. However, in ducks they usually cause an asymptomatic infection [4]. Migratory wild birds infected with HPAIVs can carry and distribute these viruses over long distances [5,6]. Vaccination of wild birds against H5N1 HPAIVs is currently not feasible, however naturally occurring infections with low-pathogenic avian influenza H5 viruses may generate cross-protective immunity against H5N1 HPAIVs [7,8,9].

Vaccination programs used so far have been primarily focused on chickens. However, in order to prevent the circulation of H5N1 HPAIVs it would be more useful to vaccinate domestic ducks, the “Trojan horses” of H5N1, that have contact both with wild aquatic birds and urban terrestrial birds [5,6,7,8,9,10].

Currently the most common HPAIV vaccines are oil-in-water emulsion adjuvanted whole virus vaccines based on reverse genetically engineered reassortants with H5 hemagglutinin (HA) and N1 neuraminidase (NA) from H5N1 viruses and the remaining genes from A/PuertoRico/8/1934 (PR8). Inactivated vaccines protect the chickens and prevent the decrease in egg production. However, such vaccines were unable to completely suppress fecal excretion of HPAIV after challenge and failed to prevent the spread of the virus due to various problems in the vaccination process and antigenic drift of field viruses from the vaccine strains [7,11,12,13]. Therefore, there is a need for development and evaluation of more effective vaccines with broader protection spectrum.

To date, several H5N1 influenza vaccines have been developed using other viruses as vectors. Such vaccines provide protection against two viral diseases. A polyvalent candidate vectored vaccine based on duck enteritis virus (DEV) with the HA gene of A/duck/Hubei/xn/2007 (H5N1) was developed by Zou et al. Ducks immunized with this live vaccine demonstrated rapid and long-lasting protection against homologous and heterologous HPAIV H5N1 and DEV [14].

Turkey herpesvirus (HVT) vector vaccine expressing the HA gene from H5N1 strain protected vaccinated birds from homologous and heterologous challenge with HPAI H5N1 and H5N2 viruses [15]. Recombinant HVT carrying a H5 (rHVT-H5) insert provided higher protection rates than an oil emulsion-adjuvanted inactivated H5 vaccine (KV-H5). The highest protection rates were obtained after rHVT-H5 and KV-H5 prime/boost vaccination [16].

Live recombinant H5N1 HPAIV vaccines have been also developed using modified Newcastle disease virus (NDV) as vector. Immunization of chickens with these NDV-vectored vaccines provided high levels of protection against clinical disease and mortality after lethal challenge with HPAIV A/Vietnam/1203/04 (H5N1) [17]. NDV-vectored H7 and H5 vaccines were able to induce high antibody levels and completely protect chickens from challenge with the novel H7N9 and highly pathogenic H5N1 viruses, respectively [18].

In addition to vectored vaccines, numerous experimental recombinant H5N1 live attenuated vaccines have been generated [19]. Vaccines were attenuated through temperature-sensitive mutations or modification of the interferon antagonist protein. These live vaccines provided protection against homologous and heterologous challenge with H5 HPAIV in animal models [20].

A series of H5N1 avian influenza virus reassortants with NS1 protein terminated at amino acids (aa) 48, 70, 73, or 99, along with a modified low pathogenic HA protein was generated, and their biological and immunological characteristics were analyzed. The recombinant virus with NS1 truncated at aa 73 was tested as a broad-spectrum live attenuated H5N1 avian influenza vaccine candidate in chickens [21].

Choi et al. constructed a dual H5N1 and H9N2 live attenuated influenza vaccine (LAIV) with HA and NA genes from an avian H5N2 virus and a chicken H9N2 virus, respectively, and A/Puerto Rico/8/34 backbone expressing truncated NS1 proteins. Such H5N2/NS1-LAIV viruses attenuated in mice were found to induce stronger IFN-β activation than viruses with full-length NS1 proteins. Intranasal inoculation with a single dose of these viruses completely protected mice from lethal challenge with heterologous highly pathogenic H5N1 and heterosubtypic highly virulent H9N2 viruses [22].

A novel live experimental H5 virus vaccine lacking neuraminidase protein was developed for early onset protection against HPAIV H5N1. A single immunization with the virus seven and three days before lethal challenge protected chickens, mice and ferrets [23].

Four reassortant viruses with modified HA genes from H5N1 clade 1 and clade 2 viruses and internal genes from a cold-adapted donor or a nonpathogenic H6N2 influenza virus were developed in our laboratory [24,25]. These experimental strains were used as live poultry vaccines in comparison with a nonpathogenic influenza virus isolated from a wild duck A/duck/Moscow/4182/2010 (H5N3) (dk/4182). The best results were obtained with the dk/4182 virus, which caused no mortality or visible signs of the disease but stimulated the development of high antibody levels and offered 100% protection against subsequent lethal challenge. Our previous studies suggest that this H5N3 wild duck virus represents a promising candidate vaccine for protection of poultry from H5N1 HPAI viruses [26,27].

In this study, we investigated whether oral administration of dk/4182 virus to ducks can prevent circulation of HPAIV H5N1 in ducks and its transmission to chickens.

## 2. Materials and Methods

### 2.1. Viruses

A/chicken/Kurgan/3/2005 (HPAI H5N1 clade 2) (ch/Ku) and A/duck/Moscow/4182/2010 (LPAI H5N3) (dk/4182) (GenBank accession numbers: DQ323672–DQ323679; KF885672–KF885679, Figure S1) were propagated in the allantoic cavity of 10-day-old embryonated special pathogen free chicken eggs at 37 °C and harvested 48 h post infection (p.i.). Infectious allantoic fluids (IAF) were pooled, divided into aliquots, and stored at −70 °C. 50% tissue culture infective dose (TCID_50_) for each virus stock was determined by titration in Madin-Darbey Canine Kidney (MDCK) cells.

### 2.2. Animals

Pekin ducks, chickens of Shaver Brown breed and embryonated chicken eggs were purchased from State poultry farm “Ptichnoe” (Moscow, Russia). All studies with HPAIV ch/Ku virus were conducted in a biosafety level 3 containment facility at the Federal Research Center for Virology and Microbiology.

### 2.3. Ethics Statement

Studies involving animals were performed in accordance with the (European Convention for the Protection of Vertebrate Animals used for Experimental and Other Scientific Purposes, Strasbourg, 18 March 1986). All appropriate measures were taken to ameliorate animal suffering.

Forty-two ducks and 177 chickens were used in the study; 38 ducks and 155 chickens survived and were subsequently kept in the bird housing facility for repeated detection of antibody levels. Four ducks and 22 chickens were humanely euthanized after they showed signs of severe disease.

The study design was approved by the Ethics Committee of the Chumakov Institute of Poliomyelitis and Viral Encephalitides, Moscow, Russia (Approval #4 from 2 December 2014).

### 2.4. Oral Administration of the Viruses to Ducks and Chickens

After leaving the birds overnight without water, one drinking bowl per 3 birds was placed in each cage in the morning. Each bowl contained 400 mL (for ducks) or 50 mL (for chickens) of diluted IAF. Individual uptake of virus-containing drinking water was not determined.

### 2.5. Challenge with HPAIV ch/Ku Virus

Aerosol challenge of chickens was performed as described previously [27]. Briefly, the birds were placed into a 200 L transparent plastic chamber connected to the Micropump Nebulizer (Aerogen, Ireland) generating virus aerosol. The aerosol entered the chamber through the inlet in its upper lid and was exhausted trough the outlet in the bottom part of the chamber connected via a high efficiency particulate arresting (HEPA) filter. Challenge of ducks and primary administration of chickens and ducks with ch/Ku virus were performed orally, as described above.

### 2.6. Virus Transmission from Ducks to Chickens

The birds were kept in two-level wire cages with the upper and lower sections separated by a metal plate that prevented feces and waste from falling to the lower section. The trays were covered with floor matting which was replaced daily. The ducks were kept in the upper section. Two days after infection of the ducks with influenza virus, the chickens were placed in the lower section and the plate separating the sections was replaced with a shorter tray covering a half of the mesh floor. Thereby, one part of the lower section remained clean, and another part, where feeders and drinking bowls were located, was contaminated with duck feces. The birds were monitored daily. Feces were sampled on days 3 to 12.

### 2.7. Detection of Influenza Viruses in Feces and Internal Organs of the Birds

Feces were sampled once daily. Clean matting was placed inside a clean tray of each cage and all fresh fecal samples were collected after 30 min. Samples of internal organs or feces were homogenized and suspended in two volumes of phosphate buffered saline supplemented with 0.4 mg/mL gentamycin, 0.1 mg/mL kanamycin, 0.01 mg/mL amphotericin B and 2% MycoKill AB (PAA Laboratories GmbH, Pasching, Austria) and centrifuged at 4000 rpm. The supernatant was used to inoculate 10-day-old embryonated chicken eggs (0.2 mL per egg). After incubation for 48 h allantoic fluids were collected and tested by hemagglutination assay with chicken red blood cells.

### 2.8. Identification of Influenza Virus by PCR

Total RNA was isolated from tissue samples by TRI Reagent LS (Sigma, T3934, St. Louis, MO, USA) and 1-Bromo-3-chlorpropane (B9673, Sigma-Aldrich) according to manufacturer’s protocol. After reverse transcription with Moloney murine leukemia virus reverse transcriptase (M1701, Promega, Madison, WI, USA) polymerase chain reaction with Taq-polymerase (Promega) was performed using specific primers for H5 HA (H840f 5′-gctccagaatatgcatacaa-3′ and H1126r 5′-tgagtgttcattttgtcaat-3′) and NP genes (NP1019f 5′-aatgagaatccagcacataa-3′ and NP1565r 5′-agtagaaacaagggtatttttc-3′). Amplicons were visualized by electrophoresis in 1.5% agarose gel in the presence of ethidium bromide.

### 2.9. Assessment of Antibody Titers

The levels of antibodies (AB) in sera were assessed by hemagglutination-inhibition assay (HAI) and by ELISA with horseradish peroxidase labeled anti-chicken IgY antibodies (Sigma-Aldrich) using dk/4182 virus as described in detail elsewhere [24]. For immunogenicity assessment the antibody levels were determined in fresh sera samples on day 14 after the primary infection. For the assessment of the duration of antibody response frozen sera samples from day 14 and 90 after the primary infection and from days 14 and 360 after the challenge were tested in ELISA simultaneously.

## 3. Results

The goal of this work was to determine whether a non-pathogenic virus of wild ducks could be used as a live poultry vaccine against HPAIVs preventing transmission of these viruses from ducks to chickens.

### 3.1. Pathogenicity and Fecal Excretion of ch/Ku and dk/4182 after Oral Administration

To study the pathogenicity of dk/4182 and ch/Ku viruses for birds, chickens and ducks of different ages were orally infected with both viruses by adding IAF to the drinking water. The birds were monitored, and feces were sampled daily. Table 1 summarizes the results of two independent experiments with ducks and three experiments with chickens.

Upon initial infection ch/Ku virus caused torticollis and sometimes death in 7-day-old ducklings and killed the chickens of all ages, but it was safe for 40-days-old ducks. The dk/4182 virus caused no signs of disease in both ducks and chickens of any age (Table 1).

Ducks and chickens infected with ch/Ku or dk/4182 excreted the viruses with feces on days 3 to 10 post infection. Virus detection rates in these groups were from 30% to 80%.

### 3.2. Immunogenicity and Protectivity of dk/4182 after Oral Administraion to Ducks and Chickens

The levels of antibodies were determined on day 14 after the primary infection. All adult chickens and ducks that received 10^6^ TCID_50_ of the dk/4182 virus via drinking water had high levels of antibodies. Antibody response to immunization with a lower dose of dk/4182 (10^5^ TCID_50_) in chickens was much lower, which corresponds to our previous data [27]. In contrast to chickens, ducks demonstrated approximately the same antibody responses after infection with 10^4^–10^6^ TCID_50_ of dk/4182 (Table 2).

To study the protectivity of dk/4182, all ducks and all grown-up chickens previously infected with dk/4182 were challenged with the HPAIV ch/Ku. The chickens were challenged via aerosol treatment 20 or 85 days following the initial administration of dk/4182, whereas the ducks were challenged with ch/Ku orally 16 or 90 days after dk/4182 administration. The oral method of challenge for ducks was selected to bring the experimental conditions closer to natural ones.

Previous infection with dk/4182 protected chickens and 7-day-old ducklings against challenge with ch/Ku (Table 1, row 3, 11). Even on day 85 after single oral administration of 10^6^ TCID_50_ dk/4182 17 of 19 chickens were protected against challenge with HPAIV ch/Ku (Table 1, row 12).

Feces of ducks and chickens were sampled daily after the challenge and the ch/Ku virus was never detected in feces of birds infected with the dk/4182 and challenged later with the ch/Ku (Table 1).

### 3.3. Duration of Antibody Response in Ducks Following Immunisation and Challenge

**Figure 1:**
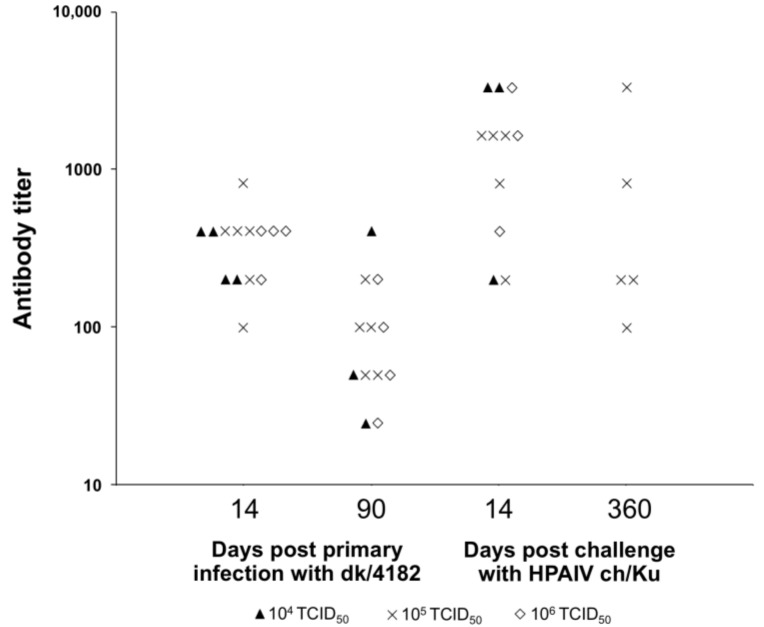
Titers of anti-H5 antibodies in sera of individual ducks (measured by ELISA) on days 14 and 90 after oral infection with 10^4^–10^6^ 50% tissue culture infective doses of dk/4182 and on days 14 and 360 after challenge with ch/Ku.

Levels of antibodies in ducks infected with 10^4^–10^6^ TCID_50_ dk/4182 at the age of 40 days were determined on day 14 after the primary infection, on the day of the challenge (90 days post primary infection) and on days 14 and 360 after the challenge. 90 days after the primary infection the antibody levels in ducks decreased but remained detectable. Following challenge with the HPAI ch/Ku virus the anti-H5 antibody levels increased significantly and remained detectable for at least one year (Figure 1).

### 3.4. Transmission of dk/4182 and ch/Ku Viruses from Ducks to Chickens

The ducks infected with dk/4182 or ch/Ku were also used to study the transmission of HPAIV from ducks to chickens. Naïve chickens were placed in the lower section of the cages 3 days after infection of the ducks. Thus, feeders and drinking bowls of the chickens were contaminated with fresh feces of the ducks infected with dk/4182 or ch/Ku. The contact chickens were monitored daily, their feces were collected from day 3 after the first exposure to feces of infected ducks. Blood samples were taken from the chickens that survived by day 14 to determine antibody levels. The internal organs of dead chickens were taken for virus detection and isolation.

Even though the dk/4182 virus was excreted with feces of ducks, chickens that came in contact with infected ducks apparently were not infected as they did not excrete the virus and did not develop antibodies to dk/4182 virus (Table 1 and Table 3). These results can be explained by the fact that a very high dose of dk/4182 virus is required for successful infection of chickens [27].

In contrast, all chickens that came in contact with the ducks primarily infected with ch/Ku, died. The presence of H5N1 virus in the internal organs of contact chickens had been proven by PCR; moreover, the virus was isolated in chicken embryos. Before dying, the contact chickens also excreted the HPAIV H5N1 in feces (Table 3).

### 3.5. Prevention of ch/Ku Transmission from Ducks to Chickens by Immunization of Ducks with the dk/4182 Virus

Ducks were challenged with ch/Ku orally 16 or 90 days after primary infection with dk/4182. Naïve chickens were placed in the lower section of the duck cages 3 days after administration of the challenge virus to ducks. The chickens that contacted the challenged ducks did not show any signs of the disease, did not excrete the virus with feces and developed no antibody response to the H5N1 virus (Table 3). Obviously, the ducks inoculated with the dk/4182 and later challenged with the ch/Ku did not excrete the ch/Ku virus with feces (Table 1).

## 4. Discussion

We have previously reported that the live LPAI H5N3 virus dk/4182 was safe for mice and chickens and provided protection against HPAI H5N1 virus challenge, while ch/Ku showed extremely high virulence in mice and chickens [25,26,27]. Here we compared pathogenicity of these viruses for ducks and chickens, modeled transmission of HPAIV H5N1 between ducks and chickens and prevented the transmission by vaccinating ducks with the dk/4182 virus.

Currently the outbreaks of the HPAIV viruses are mainly controlled by culling all poultry in the affected regions. Another way to combat highly pathogenic avian influenza viruses is vaccination of poultry. Unfortunately, vaccination programs failed to prevent worldwide spread of HPAIV H5N1. The most commonly used inactivated vaccines successfully protect layers and broiler chickens, but fail to prevent the spread of the virus. It is generally recognized that live vaccines are capable of broader protection and are superior to inactivated vaccines in preventing the circulation of viruses. Still, live vaccines against H5N1 viruses are not approved for use anywhere outside China. Potential risk of pathogenicity restoration is the main reason for strong regulatory barriers against live poultry vaccines against avian influenza viruses [28]. It is believed that reassortment with field strains or reverse mutations could restore the virulence of a vaccine virus.

Considering the virulence of wild duck influenza viruses, some researchers tried to use the genes of these viruses for the development of live influenza vaccines [21,22,24,25,26,27,29,30]. The concept of using wild duck viruses as live poultry vaccines is complicated by the fact that all HPAI viruses emerge from LPAIVs [31]. However, emergence of new HPAIV viruses is a rare phenomenon. Wild bird influenza viruses do not cause disease in their natural hosts. Host and virus exist in a state of a mutual tolerance. The viruses are evolutionary stable, and their genomes evolve with low non-synonymous vs. synonymous mutation ratios which indicate a purifying selection [32,33]. Before wild duck viruses become pathogenic, they usually circulate in chickens as LPAIVs for a long time. Indeed, it had been shown previously that adaptation of wild duck viruses to chickens and acquisition of virulence require multiple passages through air sacs [34]. It is likely that the risk of emergence of pathogenicity in a wild duck virus is lower than the risk of pathogenicity restoration for artificially constructed vaccine strains. Indeed, influenza viruses of wild birds had been subjected to natural selection towards non-virulence for centuries, while the safety of attenuated strains relies on the presence of few introduced mutations.

Dk/4182 is a typical duck virus and all its genes are in evolutionary clades containing exclusively low pathogenic viruses of wild aquatic birds (Figure S2). It replicates very well in ducks, but successful infection of chickens requires a very high dose of the virus. The virus is unable to circulate in chickens as it failed to transmit from directly inoculated ducks and chickens to contact chickens in our previous work [27].

One can speculate that occasional adaptation of the dk/4182 virus to chickens and its spread in chicken population with potential subsequent acquisition of virulence is not more likely than emergence of a new lineage of chicken viruses from freely circulating LPAIVs.

LPAI H5N3 virus dk/4182 offers substantial benefits compared to reassortants with modified HA genes from H5N1 and internal genes from a cold-adapted donor or a nonpathogenic H6N2 influenza virus [26]. The balance between pathogenicity and immunogenicity of dk/4182 was more beneficial than in the artificially generated reassortants. Dk/4182 is safe for chickens of any age, regardless of inoculation method. The virus is also non-pathogenic for mice and likely other mammals. It can be administered via drinking water [26,27].

A single oral immunization of ducks with a low dose of the live virus is sufficient to achieve prolonged sterile immunity. Oral administration and replication in intestinal epithelial cells mimic the natural infection route of avian influenza viruses and elicit a well-developed local and humoral immune response.

It is important to note that prevention of fecal excretion of the virus after challenge was the most pronounced effect of immunization. Protection from lethal challenge with HPAIV ch/Ku and prevention of its shedding with feces was achieved both by preceding direct oral administration of LPAIV dk/4182 and by contact with feces of ducks infected with dk/4182.

Since the dk/4182 virus is antigenically equidistant from all HPAI H5 viruses including the virus that was used for the challenge, it could be expected that it will be effective against a broad range of H5 HPAIV.

Our data confirm that oral vaccination of ducks with a wild duck virus can prevent the circulation of HPAIV H5N1 in ducks and its transmission from ducks to chickens. For these reasons, dk/4182 H5N3 virus represents a promising live candidate vaccine for protection of poultry from H5N1 HPAI viruses.

## Figures and Tables

**Table 1:** Survival rates and fecal excretion of viruses ch/Ku and dk/4182 following primary administration to ducks and chickens via drinking water and after aerosol challenge with ch/Ku.

№	Species	Primary Virus Administration					Challenge with 10^5^ TCID_50_^a^ ch/Ku		
		Age, Days	Virus	TCID_50_	Survival ^b^	Excretion ^c^	DPI ^d^	Survival ^b^	Excretion ^c^
1	Ducks	7	ch/Ku	10^5^	2/6	19/34			
2	Ducks	40	ch/Ku	10^5^	5/5	13/29			
3	Ducks	7	dk/4182	10^6^	6/6	16/31	16	6/6	0/18
4	Ducks	40	dk/4182	10^4^	8/8	15/20	90	8/8	0/17
5	Ducks	40	dk/4182	10^5^	10/10	17/22	90	10/10	0/23
6	Ducks	40	dk/4182	10^6^	7/7	15/18	90	7/7	0/19
7	Chickens	7	ch/Ku	10^5^	0/5	7/20			
8	Chickens	50	ch/Ku	10^5^	0/5	13/25			
9	Chickens	1	dk/4182	10^6^	6/6	4/10			
10	Chickens	7	dk/4182	10^6^	10/10	8/18			
11	Chickens	30	dk/4182	10^6^	80/80	22/67	20	25/25	0/47
12	Chickens	27	dk/4182	10^6^	30/30	19/42	85	17/19	0/32

^a^ TCID_50_: 50% tissue culture infective dose. ^b^ Survived/total number used for primary virus administration. ^c^ Excretion of the ch/Ku or dk/4182 virus with feces on days 3 to 12 after last administration of the viruses. Positive/total number of samples. ^d^ DPI: days post primary dk/4182 virus administration.

**Table 2:** Levels of antibodies following primary oral administration of dk/4182 to ducks and chickens.

№	Species (Number)	Age, Days	Virus	Dose ^a^	ELISA ^b^	HAI ^c^
**1**	Ducks (8)	40	dk/4182	10^4^	249	26
**2**	Ducks (5)	40	dk/4182	10^5^	228	28
**3**	Ducks (7)	40	dk/4182	10^6^	255	17
**4**	Chickens (5)	30	dk/4182	10^5^	193	11
**5**	Chickens (10)	30	dk/4182	10^6^	463	37

^a^ Virus dose, TCID_50_/bird. ^b^ Geometric mean titer of antibodies measured by ELISA. ^c^ Geometric mean titer of antibodies measured by HAI assay.

**Table 3:** Survival, fecal excretion of the virus and antibody responses in chickens that contacted the ducks primarily infected with dk/4182 or ch/Ku.

Number of Ducks	Primary Infection of Ducks			Challenge ^a^	Infection Rate in Contact Chickens		
	Age, Days	Virus	TCID_50_	DPI ^b^	Survival ^c^	Excretion ^d^	ELISA ^e^
Transmission after primary administration							
6 ^f^	7	dk/4182	10^6^		5/5	0/25	<10
6	7	ch/Ku	10^5^		0/5	12/23	N/A
5	40	ch/Ku	10^5^		0/5	5/9	N/A
Transmission after challenge with ch/Ku							
6 ^f^	7	dk/4182	10^6^	16	7/7	0/27	<10
5	40	dk/4182	10^4^	90	5/5	0/11	<10
5	40	dk/4182	10^5^	90	5/5	0/9	<10
4	40	dk/4182	10^6^	90	4/4	0/13	<10

^a^ Challenge with 10^5^ TCID_50_ ch/Ku. ^b^ DPI—days post primary dk/4182 virus administration. ^c^ Survived/total number. ^d^ Excretion of the ch/Ku or dk/4182 virus with chicken feces. Positive/total number of samples. ^e^ AB—Geometric mean titer of antibodies measured by ELISA. ^f^ 6 ducks were primarily infected with dk/4182 and later challenged with ch/Ku; the numbers of contact chickens in the rows represent two different experiments. <10—Below detection limit of the assay. N/A—Not available.

## Supplementary Materials

The following are available online at http://www.mdpi.com/1999-4915/10/4/164/s1, Figure S1: Influenza Strain Details for A/duck/Moscow/4182/2010(H5N3), Figure S2: Phylogenetic relationships of A/duck/Moscow/4182/2010 (H5N3).
