# Supplementary material for: Immunization of Domestic Ducks with Live Nonpathogenic H5N3 Influenza Virus Prevents Shedding and Transmission of Highly Pathogenic H5N1 Virus to Chickens
Source: Viruses. 2018 Mar 31;10(4):164. doi: 10.3390/v10040164 (PMC5923458; doi:10.3390/v10040164)
Supplement: Supplementary file 1 [file viruses-10-00164-s001.zip › Supplementary/Figure S1.pdf]

Influenza Strain Details for A/duck/Moscow/4182/2010(H5N3)

Strain Information

Strain Name	**IRD:**A/duck/Moscow/4182/2010(H5N3)<br>**GenBank:**A/duck/Moscow/4182/2010
Organism Name	*Influenza A Virus*
Subtype	H5N3
Host	**IRD:**Duck/Avian<br>**GenBank:**wild duck
2009 Pandemic H1N1-like (SOP) ?	Negative
Isolation Country	Russia
Collection Date	11/2010
Season	10-11
GenBank Submission Date	02/06/2014
Isolation Source	feces
NCBI Taxon ID	1428187
Complete Genome Set	Yes

Sequence Information

	Segment	Subtype	Gene Product Name	GenBank Source Sequence Accession	Complete Sequence	Segment Length	IRD Submission	pH1N1-like
View	1	H5N3	PB2 Polymerase (basic) protein 2	KF885672	Complete	2329	-N/A-	No
View	2	H5N3	PB1 Polymerase (basic) protein 1, PB1-F2	KF885673	Complete	2329	-N/A-	No
View	3	H5N3	PA Polymerase (acidic) protein, PA-X protein(+61)	KF885674	Complete	2214	-N/A-	No
View	4	H5N3	HA Hemagglutinin	KF885675	Complete	1737	-N/A-	No
View	5	H5N3	NP Nucleoprotein	KF885676	Complete	1540	-N/A-	No
View	6	H5N3	NA Neuraminidase	KF885677	Complete	1453	-N/A-	No
View	7	H5N3	M1 Matrix protein 1, M2 Matrix protein 2	KF885678	Complete	1027	-N/A-	No
View	8	H5N3	NS1 Non-structural protein 1, NS2 Non-structural protein 2	KF885679	Complete	874	-N/A-	No

Sequence Derived Phenotype Marker

Phenotype	Substitution Type	Present
Adamantane Resistance	M2 A30T	No
Adamantane Resistance	M2 G34E	No
Adamantane Resistance	M2 L26F	No
Adamantane Resistance	M2 S31N (well characterized)	No
Adamantane Resistance	M2 V27A	No
Enhanced Transmission	M2 A16G	No
Enhanced Transmission	M2 C55F	No
Enhanced Transmission	NP L136M	No
Enhanced Transmission	PA S409N	No
Enhanced Transmission	PB2 A199S	No
Enhanced Transmission	PB2 A661T	No
Enhanced Transmission	PB2 K702R	No
Enhanced Transmission	PB2 V667I	No
Increased Virulence	PB1-F2 N66S	No
Increased Virulence	PB2 D701N	No
Increased Virulence	PB2 E627K	No
Polybasic HA Cleavage	RERRKKR	No
Severity	NS1 T92E	No

Data was obtained from the NIAID IRD online through the web site at http://www.fludb.org
