# Supplementary material for: Immunization of Domestic Ducks with Live Nonpathogenic H5N3 Influenza Virus Prevents Shedding and Transmission of Highly Pathogenic H5N1 Virus to Chickens
Source: Viruses. 2018 Mar 31;10(4):164. doi: 10.3390/v10040164 (PMC5923458; doi:10.3390/v10040164)
Supplement: Supplementary file 1 [file viruses-10-00164-s001.zip › Supplementary/Figure S2.doc]

**Phylogenetic relationships of A/duck/Moscow/4182/2010 (H5N3)**

**Designation:** filled blue circle – apathogenic strain isolated in Russia, empty blue circle – strains with genotype similar to А/duck/Moscow/4182/2010(H5N3) that has genotype [G,G,D,5G,F,3B,F,1E] (PB2,PB1,PA,HA,NP,NA,M,NS, respectively [6]). Filled red triangle - highly pathogenic influenza virus. Empty red triangle – low pathogenic influenza virus from wild birds with HA connecting peptide of apathogenic phenotype.

**Figure 1. Evolutionary relationships of HA**

The evolutionary history was inferred using the Minimum Evolution method [1]. The optimal tree with the sum of branch length = 0.53785019 is shown. The percentage of replicate trees in which the associated taxa clustered together in the bootstrap test (1000 replicates) are shown next to the branches [2]. The tree is drawn to scale, with branch lengths in the same units as those of the evolutionary distances used to infer the phylogenetic tree. The evolutionary distances were computed using the p-distance method [3] and are in the units of the number of base differences per site. The ME tree was searched using the Close-Neighbor-Interchange (CNI) algorithm [3] at a search level of 2. The Neighbor-joining algorithm [4] was used to generate the initial tree. The analysis involved 37 nucleotide sequences. All positions containing gaps and missing data were eliminated. There were a total of 1673 positions in the final dataset. Evolutionary analyses were conducted in MEGA5 [5].

***Figure 2*. Inferred Ancestral Sequences of NA**

Ancestral states were inferred using the Maximum Likelihood method [1] under the Tamura-Nei model. The tree shows a set of possible nucleotides (states) at each ancesteral node based on their inferred likelihood at site 1. The set of states at each node is ordered from most likely to least likely, excluding states with probabilities below 5%. The initial tree was infered using a pre-computed tree file. The rates among sites were treated as being uniform among sites (Uniform rates option). The analysis involved 27 nucleotide sequences. All positions containing gaps and missing data were eliminated. There were a total of 1394 positions in the final dataset. Evolutionary analyses were conducted in MEGA5 [5].

PB2

**Figure 3. Evolutionary relationships of PB2**

The evolutionary history was inferred using the Minimum Evolution method [1]. The optimal tree with the sum of branch length = 1.27075509 is shown. The percentage of replicate trees in which the associated taxa clustered together in the bootstrap test (1000 replicates) are shown next to the branches [2]. The tree is drawn to scale, with branch lengths in the same units as those of the evolutionary distances used to infer the phylogenetic tree. The evolutionary distances were computed using the p-distance method [3] and are in the units of the number of base differences per site. The ME tree was searched using the Close-Neighbor-Interchange (CNI) algorithm [3] at a search level of 2. The Neighbor-joining algorithm [4] was used to generate the initial tree rooted relative to A/turkey/Kansas/4880/1980 (H1N1). The analysis involved 87 nucleotide sequences. All positions containing gaps and missing data were eliminated. There were a total of 2238 positions in the final dataset. Evolutionary analyses were conducted in MEGA5 [5].

**_____________________________________________________________**

PB1

**Figure 4. Evolutionary relationships of PB1**

The evolutionary history was inferred using the Minimum Evolution method [1]. The ME tree was searched using the Close-Neighbor-Interchange (CNI) algorithm [3] at a search level of 2. The Neighbor-joining algorithm [4] was used to generate the initial tree rooted relative to A/turkey/Kansas/4880/1980 (H1N1). The analysis involved 87 nucleotide sequences. All positions containing gaps and missing data were eliminated. There were a total of 2259 positions in the final dataset. Evolutionary analyses were conducted in MEGA5 [5].

**___________________________________________________________________**

PA

**Figure 5. Evolutionary relationships of PA**

The evolutionary history was inferred using the Minimum Evolution method [1]. The ME tree was searched using the Close-Neighbor-Interchange (CNI) algorithm [3] at a search level of 2. The Neighbor-joining algorithm [4] was used to generate the initial tree rooted relative to A/Iova/1943(H1N1) (H1N1). The analysis involved 89 nucleotide sequences. All positions containing gaps and missing data were eliminated. There were a total of 2140 positions in the final dataset. Evolutionary analyses were conducted in MEGA5 [5].

___________________________________________________________________________

**Figure 6. Evolutionary relationships of NP**

The evolutionary history was inferred using the Minimum Evolution method [1]. The optimal tree with the sum of branch length = 1.20518809 is shown. The percentage of replicate trees in which the associated taxa clustered together in the bootstrap test (1000 replicates) are shown next to the branches [2]. The tree is drawn to scale, with branch lengths in the same units as those of the evolutionary distances used to infer the phylogenetic tree. The evolutionary distances were computed using the p-distance method [3] and are in the units of the number of base differences per site. The ME tree was searched using the Close-Neighbor-Interchange (CNI) algorithm [3] at a search level of 2. The Neighbor-joining algorithm [4] was used to generate the initial tree. The analysis involved 95 nucleotide sequences. All positions containing gaps and missing data were eliminated. There were a total of 1432 positions in the final dataset. Evolutionary analyses were conducted in MEGA5 [5].

______________________________________________________________-

**Figure 7.** **Evolutionary relationships of M.** The tree of M segment was constructed using the Minimum Evolution method.

**Figure 8. Evolutionary relationships of NS.** The tree of NS segment was constructed using the Minimum Evolution method with interior-branch and bootstrap test (n=1000) for 84 nucleotide sequences with length of 832 nucleotides. Only branch of NS subtype 1 is shown.

**References**

1. Rzhetsky A. and Nei M. (**1992**). A simple method for estimating and testing minimum evolution trees.*Molecular Biology and Evolution* **9**:945-967.

2. Felsenstein J. (**1985**). Confidence limits on phylogenies: An approach using the bootstrap. *Evolution***39**:783-791.

3. Nei M. and Kumar S. (**2000**). *Molecular Evolution and Phylogenetics*. Oxford University Press, New York.

4. Saitou N. and Nei M. (**1987**). The neighbor-joining method: A new method for reconstructing phylogenetic trees. *Molecular Biology and Evolution* **4**:406-425.

5. Tamura K., Peterson D., Peterson N., Stecher G., Nei M., and Kumar S. (**2011**). MEGA5: Molecular Evolutionary Genetics Analysis using Maximum Likelihood, Evolutionary Distance, and Maximum Parsimony Methods. *Molecular Biology and Evolution* 28: 2731-2739.

6. FluGenome: a web tool for genotyping influenza A virus G. Lu; T. Rowley; R. Garten; R. O. Donis Nucleic Acids Research 2007; <http://flugenome.org/>
